# Fast-Track Aspirin Odyssey: ICU Chronicles

**DOI:** 10.7759/cureus.70327

**Published:** 2024-09-27

**Authors:** Malay Rathod, Shivani Modi, Sai Gaddameedi, Urja Mehta, Sarkar Sohini

**Affiliations:** 1 Internal Medicine, Monmouth Medical Center, Rutgers University, Long Branch, USA; 2 Internal Medicine, Jefferson Einstein Healthcare Network, Norristown, USA; 3 Internal Medicine, Our Lady of Fatima University, Manila, PHL

**Keywords:** aspirin, aspirin allergy, aspirin desensitization protocols, cardiac catheterization and stent placement, long term follow up, medical icu

## Abstract

Aspirin is used in patients with coronary artery disease essential in both acute and chronic phases of treatment, especially post-catheterization and post-coronary artery stent placement. Some patients have sensitivity to aspirin. Hypersensitivity reaction symptoms include itchy and watery eyes, itchy rash, worsening asthma, wheezing to fatal angioedema, and anaphylaxis. In such cases, clopidogrel can be used instead of aspirin if it is necessary to avoid the use of aspirin. Alternatively, we can try desensitization to aspirin. In aspirin desensitization, incremental doses of aspirin are provided at fixed time intervals. It usually lasts between one and three days. These protocols are often impractical in emergent conditions, especially in conditions where percutaneous coronary intervention (PCI) reveals coronary artery stenosis requiring stent placement. Post-stent placement long-term treatment with aspirin is needed. This has led to limited application in clinical practice despite the potential benefits. We present a case of a patient who presented to us with complaints of shortness of breath and intermittent chest pain. A thorough evaluation was conducted, including cardiac catheterization, which revealed a 70% blockage in the right coronary artery (RCA) and a 65% blockage in the left anterior descending (LAD) artery, necessitating stent placement. The patient reported a severe allergy to aspirin, requiring aspirin desensitization. Rapid aspirin desensitization was successfully performed in the ICU, taking two hours and 15 minutes. The patient underwent PCI and stent placement in the RCA the following day. She is currently on dual antiplatelet therapy with aspirin and clopidogrel and has scheduled follow-ups with both a cardiologist and an allergist.

## Introduction

Aspirin remains the cornerstone of therapy for patients with confirmed atherosclerotic cardiovascular disease (ASCVD), used both to manage acute events and to prevent recurrences [1]. In patients with established atherosclerotic disease, whether involving the cerebral, coronary, or peripheral arteries, aspirin is recommended as a first-line treatment alongside lifestyle modifications. It should be initiated as soon as possible in acute ischemic events and continued for life.

Aspirin's importance is particularly pronounced in patients with acute coronary syndromes undergoing percutaneous coronary interventions (PCI). Dual antiplatelet therapy (DAPT) is essential in such cases, with the only guideline-approved regimen being the combination of aspirin with a P2Y12 inhibitor.

Unlike other cardiovascular drug classes, which have several alternatives (e.g., statins, beta-blockers, and antiplatelet agents), aspirin is unique in its ability to selectively and irreversibly block platelet cyclooxygenase-1 (COX-1), providing antithrombotic benefits unmatched by other non-steroidal anti-inflammatory drugs (NSAIDs) [2].

However, aspirin is frequently discontinued due to intolerance. The National Institute for Clinical Excellence (NICE) in the United Kingdom defines aspirin intolerance as either proven hypersensitivity or a history of severe indigestion caused by low-dose aspirin [3]. Intolerance affects 6-20% of the population, with true hypersensitivity occurring in 0.6-2.4%. Although aspirin allergy is reported in 1.5-2.6% of patients with coronary heart disease (CHD), most cases are not true immune-mediated allergies but rather intolerance related to aspirin's mechanism of action [4].

Distinguishing between types of aspirin hypersensitivity and managing these patients remains a significant challenge in modern cardiology. This challenge has not been fully addressed by recent therapeutic advancements.

## Case presentation

Our patient is a 59-year-old female with active medical issues, including diabetes and hypertension, who presented to the emergency department (ED) for the evaluation of intermittent chest pain and shortness of breath for two days. She complained of midsternal left-sided chest pain radiating to the left shoulder on exertion, associated with dyspnea, diaphoresis, and dizziness. She denied fever, nausea, vomiting, leg swelling, recent viral illness, or recent travel. Family history was significant for myocardial infarction in her father before 50 years of age.

**Figure 1:**
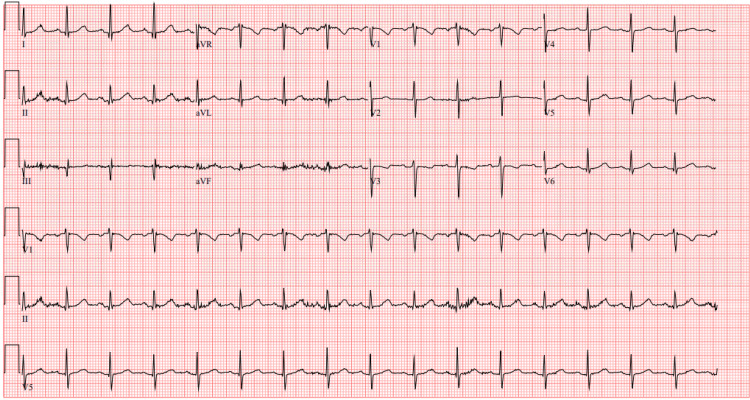
Initial EKG suggesting normal sinus rhythm EKG: electrocardiogram

**Figure 2:**
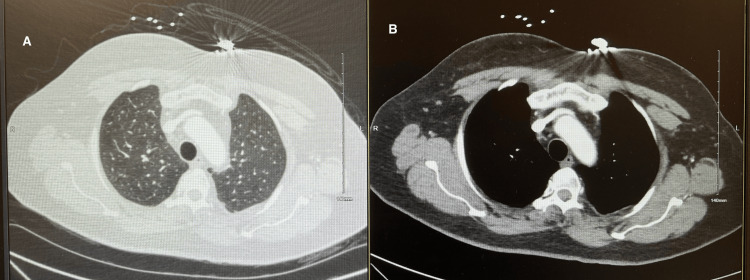
CT angiography of the chest suggesting no evidence of pulmonary embolism (A) CT angiography of the chest in the lung window suggesting no evidence of pulmonary embolism. (B) CT angiography of the chest in the mediastinal window suggesting no evidence of pulmonary embolism

The patient was hemodynamically stable with normal sinus rhythm on electrocardiogram (EKG) as shown in Figure 1. Labs were within normal limits, including negative troponin levels. The initial lab results are provided in Table 1. No significant findings were noted on physical examination. To evaluate the cause of chest pain, a battery of tests were performed. She underwent an exercise stress test but desaturated to 87% oxygen saturation (SpO2), so a pharmacological stress test was performed. No ischemic changes were noted on the EKG during the stress test. To evaluate the cause of acute-onset dyspnea during the exercise stress test, a pulmonologist was consulted. The six-minute walk test was negative, and the CT angiography of the chest for pulmonary embolism was also negative as shown in Figure 2. The echocardiogram showed an ejection fraction of 60-65% without any wall motion abnormality. The echocardiogram with bubble study for the right-to-left shunt was negative. Ultimately, cardiac catheterization was performed, which demonstrated 70% blockage in the right coronary artery (RCA) and 65% blockage in the left anterior descending (LAD) artery requiring stent placement. Of note, our patient reported a severe allergy to aspirin presenting as throat closure, difficulty in breathing, hives, and facial swelling. An allergist was consulted and she was transferred to the ICU to initiate an aspirin desensitization protocol.

**Table 1:** Initial lab values of the patient CO2: carbon dioxide; BUN: blood urea nitrogen; eGFR: estimated glomerular filtration rate; ALT: alanine aminotransferase; AST: aspartate aminotransferase; WBC: white blood cells; RBC: red blood cells; TSH: thyroid-stimulating hormone; BNP: brain natriuretic peptide; FEU: fibrinogen equivalent units

Laboratory test	Patient's result	Reference range
Serum sodium	149 mmol/L	135-145 mmol/L
Serum potassium	4.4 mmol/L	3.5-5.2 mmol/L
Serum chloride	102 mmol/L	99-109 mmol/L
Serum CO2	27 mmol/L	24-35 mmol/L
Anion gap	11 mmol/L	5-15 mmol/L
Serum BUN	12 mg/dL	5-21 mg/dL
Serum creatinine	0.63 mg/dL	0.4-1.1 mg/dL
eGFR	>60 mL/min/1.73 m^2^	>60 mL/min/1.73 m^2 ^
Serum ALT	41 U/L	10-43 U/L
Serum AST	33 U/L	13-41 U/L
Serum albumin	4.9 g/dL	3.5-5 g/dL
Serum alkaline phosphatase	124 U/L	42-119 U/L
Serum total bilirubin	0.8 mg/dL	0.2-1.2 mg/dL
Serum calcium	9.7 mg/dL	8.3-10.2 mg/dL
Serum glucose	186 mg/dL	70-110 mg/dL
Serum magnesium	1.53 mg/dL	1.5-2.5 mg/dL
Serum phosphorus	4.3 mg/dL	2.3-4.5 mg/dL
Serum total protein	7.4 g/dL	6.4-8.3 g/dL
WBC	7.9 10^3^/uL	4.50-11.00 10^3^/uL
RBC	4.25 10^3^/uL	4.20-5.40 10^6^/uL
Hemoglobin	12.4 g/dL	12-16 g/dL
Platelet	291 10^3^/uL	140-450 10^3^/uL
Troponin I high sensitivity	0.004 ng/mL	0.002-0.045 ng/mL
Sedimentation rate	20 mm/hr	0-30 mm/hr
D-dimer	0.31 ug/mL FEU	≤0.500 ug/mL FEU
TSH	1.168 UIU/mL	0.300-5.000 UIU/mL
C-reactive protein	1.78 mg/L	≤7.00 mg/L
Serum BNP	7 pg/mL	≤100 pg/mL

Rapid aspirin desensitization was performed in the ICU as per the protocol provided in Table 2. Vitals were checked every hour. A crash cart was stationed in her room for the management of anaphylactoid reaction. She remained hemodynamically stable and did not have any reaction to aspirin. The desensitization protocol was performed successfully within two hours and 15 minutes.

**Table 2:** Aspirin desensitization protocol. Aspirin dosages are diluted in 10 mL of sterile water

Time, in minutes	Aspirin dose, in mg
0	0.1
15	0.3
30	1
45	3
60	10
75	40
90	80
105	162
120	365

The patient underwent PCI the next day and a drug-eluting stent was placed in the RCA. Unfortunately, secondary to radial artery spasm, the instantaneous wave-free ratio (iFR) of the LAD could not be performed; thus, we were unable to determine whether LAD stenting was needed or not. She was discharged on DAPT with aspirin 325 mg and clopidogrel 75 mg daily for 30 days, with the recommendation for close follow-up with a cardiologist and allergist outpatient. Subsequently, the aspirin dosage was reduced to 81 mg daily after one month of stent placement.

## Discussion

Aspirin is a COX-1, and it acts by the acetylation of COX-1 in platelets causing the inhibition of thromboxane A2 (TXA2) synthesis. Decreased production of TXA2 causes impaired platelet aggregation [3]. Aspirin allergy is noted in 6-20% population, but true aspirin sensitivity is prevalent in only 0.6-2.4%. Symptoms of the aspirin allergy can range from rhinitis, abdominal cramping, and urticaria to fatal angioedema, hypotension, laryngeal edema, and respiratory distress [4]. DAPT including aspirin and P2Y12 inhibitors is the treatment of choice for the secondary prevention of atherothrombotic events in patients with coronary artery disease, especially those undergoing PCI with stent placement. Class 1 recommendations for PCI suggest that a patient, if not on aspirin therapy, should be given 325 mg of aspirin as soon as possible before PCI. After PCI, aspirin should be continued for an indefinite time in doses between 81 mg and 325 mg [1].

In our case, after PCI and stent placement, the patient should be on DAPT according to the guidelines. Here, it is challenging because of the history of severe allergic reaction to aspirin. Therefore, we opted to perform aspirin desensitization to initiate and continue aspirin post-PCI and stent placement. The mechanism of aspirin desensitization is not completely understood, but the process consists of small incremental dosages of aspirin causing a reduction in leukotriene production and a reduction in histamine and tryptase release from mast cells [4].

Several effective aspirin desensitization protocols exist, many of which are typically performed in outpatient settings. Wong and colleagues conducted aspirin desensitization in 11 patients with NSAID-induced urticaria, nine of whom had a history of coronary artery disease. In this protocol, dosing intervals were individualized to 10-30 minutes with a maximum dose of 325 mg [5]. Schaefer and Gore implemented a desensitization protocol over three days with a maximum dose of 650 mg [6]. Silberman and colleagues used a protocol starting with a 1 mg dose and increasing to a maximum of 100 mg, with 30-minute intervals between doses and dose doubling at each interval [7]. However, some of these protocols are very time-consuming and impractical in emergent situations, such as acute coronary syndrome, or when immediate aspirin administration is required following PCI and stent placement. Currently, there is only one prospective, multicenter, observational study that evaluated aspirin desensitization in patients with coronary artery disease, with a protocol lasting 5.5 hours and a maximum dose of 100 mg in an inpatient setting [8]. However, no standardized protocol currently exists that uses a test dose of 325 mg.

Rapid aspirin desensitization protocols should be performed in facilities with multidisciplinary support, including access to resuscitation resources and close monitoring of the patient. Once the patient has become desensitized, they must continue aspirin therapy without interruption, as a break in treatment of 1-5 days can lead to a sensitized state again risking an anaphylactoid reaction. Scheduled frequent follow-up with an allergist and cardiologist is also important.

Currently, there is no international protocol for rapid desensitization in patients with a history of coronary artery disease. We need an internationally standardized protocol for desensitization in such patients. More research with a larger cohort is required to develop rapid protocols.

## Conclusions

Diagnosing true aspirin sensitivity is challenging and often necessitates the expertise of an allergist. This sensitivity can significantly impact the initiation and adherence to aspirin therapy. Aspirin, along with P2Y12 inhibitors, is a cornerstone of management following cardiac catheterization and stent placement, and it is a class 1 recommendation. However, an allergy to aspirin complicates the initiation and maintenance of DAPT post-stent placement, making aspirin desensitization crucial. Most desensitization protocols are time-intensive and are usually conducted in an outpatient setting, which is impractical during cardiac emergencies such as acute coronary syndrome. We successfully implemented a rapid desensitization protocol in the ICU without notable complications, highlighting the need for further research into the development of such rapid protocols.
